# Palladium Membrane with High Density of Large-Angle Grain Boundaries to Promote Hydrogen Diffusivity

**DOI:** 10.3390/membranes12060617

**Published:** 2022-06-14

**Authors:** Efi Hadjixenophontos, Masoud Mahmoudizadeh, Michael Rubin, Dirk Ullmer, Fatemeh Razmjooei, Alexander C. Hanf, Jan Brien, Roland Dittmeyer, Asif Ansar

**Affiliations:** 1 German Aerospace Center, Institute of Engineering Thermodynamics, DLR, Pfaffenwaldring 38-40, 70569 Stuttgart, Germany; ehadjixenophontos@gmail.com (E.H.); dirk.ullmer@dlr.de (D.U.); fatemeh.razmjooei@dlr.de (F.R.); 2 Institute for Micro Process Engineering (IMVT), Karlsruhe Institute of Technology (KIT), Hermann-von-Helmholtz-Platz, 176344 Eggenstein-Leopoldshafen, Germany; masoud.mahmoudizadeh@kit.edu (M.M.); michael.rubin@kit.edu (M.R.); roland.dittmeyer@kit.edu (R.D.); 3 LT GASETECHNIK, Martener Str. 535, 44379 Dortmund, Germany; a.hanf@lt-gasetechnik.com (A.C.H.); jan.brien@lt-gasetechnik.de (J.B.)

**Keywords:** palladium membrane, hydrogen separation, suspension plasma spraying, vacuum plasma spraying, yttria-stabilized zirconia, porous Crofer 22 APU substrate

## Abstract

A higher density of large-angle grain boundaries in palladium membranes promotes hydrogen diffusion whereas small-angle grain boundaries suppress it. In this paper, the microstructure formation in 10 µm thick palladium membranes is tuned to achieve a submicronic grain size above 100 nm with a high density of large-angle grain boundaries. Moreover, changes in the grain boundaries’ structure is investigated after exposure to hydrogen at 300 and 500 °C. To attain large-angle grain boundaries in Pd, the coating was performed on yttria-stabilized zirconia/porous Crofer 22 APU substrates (intended for use later in an ultracompact membrane reactor). Two techniques of plasma sprayings were used: suspension plasma spraying using liquid nano-sized powder suspension and vacuum plasma spraying using microsized powder as feedstock. By controlling the process parameters in these two techniques, membranes with a comparable density of large-angle grain boundaries could be developed despite the differences in the fabrication methods and feedstocks. Analyses showed that a randomly oriented submicronic structure could be attained with a very similar grain sizes between 100 and 500 nm which could enhance hydrogen permeation. Exposure to hydrogen for 72 h at high temperatures revealed that the samples maintained their large-angle grain boundaries despite the increase in average grain size to around 536 and 720 nm for vacuum plasma spraying and suspension plasma spraying, respectively.

## 1. Introduction

Hydrogen is a key industrial chemical in fertilizers, alcohols, refineries, metal industry, etc. [1]. It can also be a clean fuel for power generation, for example, through fuel cells, in transport and stationary applications. Today, most of the hydrogen originates from reforming fossil fuels [2] and therefore cost [3,4], safety and high carbon dioxide (CO_2_) emission concerns demand decentralized production at a reasonable efficiency and cost. Membrane technology has gained much attention as membranes could operate more simply, with higher adaptability, lower weight, and more compact design compared to other conventional separation technologies. They not only have lower capital, operating, and maintenance costs but also a much lower impact on the environment [5]. Furthermore, they could be integrated with reactors into one module, so-called membrane reactors. Membrane reactors facilitate the proceeding of a reaction through the constant removal of a desired component from the retentate side to the permeate part so that it, in turn, might lead to reaching even beyond thermodynamics equilibrium [6]. With especial focus on membrane steam reformers where on-site hydrogen production–separation could be considered, the module should withstand operating conditions, e.g., rather high temperatures and pressures. Considering the requirements of a highly efficient membrane reactor, the membrane plays a vital role that is the main focus of this research. In the last few years, a vast range of membranes have been investigated in order to meet the hydrogen production needed for industry. We considered this spectrum from polymer-based membranes [7,8,9] to inorganic dense metallic ones, which are considered highly selective towards hydrogen, operate in the demanding conditions of higher temperatures and pressures and exhibit the ability to enable a single separation step with a solution-diffusion mechanism [4,10]. While many metals have been investigated (platinum (Pt), nickel (Ni), and metallic elements of groups III-V), palladium-based membranes have the highest interest because they are not only highly selective towards hydrogen [10,11,12] but they are also thermally and mechanically stable for a variety of applications.

The mechanism of hydrogen permeation through palladium follows a solution-diffusion mechanism, consisting of the dissociation of adsorbed hydrogen onto the metal surface, diffusion of atomic hydrogen through bulk metal, and associative desorption of the hydrogen from the metal surface. To date, there is a high interest in understanding whether larger grains can inhibit [13,14,15] or enhance [16,17] hydrogen flux. A possible correlation has been proposed between the solubility of hydrogen and the average grain boundary density which suggests that diffusion is practically unaffected as long as the hydrogen transport is controlled by bulk diffusion [18]. This is not always so simple, however, since introducing impurities and investigating alloys (a direction often discussed for decreasing the price of the membranes) [19], has shown the opposite effect [20]. It is well-known, nevertheless, that in pure metals, hydrogen segregates towards grain boundaries (GBs) [21,22]. Growing evidence shows that GBs play a crucial role in the overall performance of membranes and defects play an important role in the kinetics of hydrogen in metals [23].

Additionally, studies carried out on various types of grain boundaries over nickel found an enhanced hydrogen diffusion along large-angle boundaries (LAGB) [24]. This was also shown, using molecular dynamics simulations, in work by Ievlev et al. [25], who reported that the different types of GBs have a different effect of absorption activity for hydrogen. While discussing hydrogen permeation tests, Oudriss et al. [26] suggested that high-energy boundaries (consisting of large-angle boundaries—LAGB) act as fast diffusion paths while low-energy grain boundaries (consisting of low-angle boundaries) act as hydrogen trapping sites, lowering in this way the hydrogen flux. This trapping effect can weaken the metallic bond and cause intergranular fracture in the metal [27]. An effect which is also seen in body-centered cubic (BCC) [28] metals in comparison to face-centered cubic (FCC) ones [29,30]. It is not easy to give a direct relationship between grain boundaries and hydrogen flux, since many factors affect the determination. However, one can argue from the above-mentioned that, in general, an FCC metal with an increased amount of large-angle grain boundaries and nanoscale grain size is preferable as a membrane material [31].

Decreasing the grain size increases the GB and, therefore, theoretically a maximum permeation should be possible. Nanocrystalline microstructures (below 40 nm), lead to primarily GB diffusion instead of a lattice diffusion or even coexistence of both mechanisms that may improve the hydrogen permeation [23]. The high amount of free volume in GB enriches solubility, leading to faster diffusion for hydrogen and change of the rate-limiting step of the process. This effect has been observed in electrodeposited PdFe [13] and PdNi [32] alloy membranes.

An important aspect of Pd-based membranes is the α-β transition of palladium and its relation to grain size where a reduction in the grain size to nm size may suppress the palladium α-β transition [33] and increase the stability [34,35]. Moreover, the post-annealing at high temperatures of nanostructured coatings coated by chemical vapor deposition (CVD) or electroless plating (ELP) resulted in an increase in the grain size of the membrane and also an increase in diffusivity into the metallic layer and the selectivity of the hydrogen [36,37]. Guazzone and Ma [38] discussed in detail where nanograins might enhance hydrogen diffusion due to higher LAGB or mix diffusion, however, the inter-crystalline spaces also allow undesired gases to pass through, consequently reducing membrane selectivity. Moreover, they also suggested that the formation of pinholes in nanostructured palladium (Pd) when sintering due to palladium self-diffusion is significantly high. Furthermore, the formation of nanostructured Pd might result in SAGBs; consequently, suppressing the hydrogen diffusion. Another key parameter, when forming a Pd/Pd-alloy membrane, is the layer thickness. The thinner layer reduces mass transport resistance; however, thin layers need smooth substrates/diffusion barrier layer surfaces. For instance, the “pore-filled” method has been reported to form a thin layer of Pd, e.g., less than 2 µm onto an intermediate layer for Pd-based composite membranes, however, the difference between the thermal expansion coefficient of the intermediate layer and Pd is of great importance [33,39,40]. This work has been aimed at the formation of a layer (≈10 µm) on a porous substrate, as has been frequently reported [41,42].

The fabrication method influences drastically the final morphology of the membrane and resulting microstructures. In the literature, different techniques for the coating of a Pd/Pd-alloy could be found for which the pros and cons are discussed briefly here for the most common ones. The advantages of ELP could be the simplicity of the equipment needed for coating, its cheapness, and applicability for any membrane shape [43]. On the other hand, this technique requires several preparation steps such as activation and sensitization and it is also time consuming. In comparison with ELP, the method of CVD facilitates the control of film formation so that thinner films, e.g., <2 µm are often obtained. However, it requires Pd precursors of high volatility and high thermal stability for high yield and short processing times [44,45,46]. Another technique to be addressed is physical vapor deposition that provides the user with a good control of the thickness, phase and composition of the film. As drawbacks, this technique needs expensive equipment and it also might be limited to only flat geometries [47,48].

Combining the requirements of good hydrogen permeability and retaining sufficient selectivity remains a fundamental challenge for the fabrication processes [49]. This work attempts to produce a thin submicronic microstructured Pd through a promising cost and time-efficient production method based on industrially established plasma spraying techniques. In this case, it takes only minutes to deposit the 10–20 µm coating and substrates need no activation or other special treatment before use [50,51].

Plasma spraying typically uses metallic feedstock powders of particles size typically 20–60 µm and often leads to thicker palladium membranes in the range of 45–60 mm [52,53] which give unsatisfactory poor permeability due to high thickness but also, sometimes, poor selectivity towards hydrogen due to porosity, and the instability of the film. For thinner coatings, using a nanosized powder feedstock is one option. Dry powders of nanosize, however, lead to an agglomeration of particles, amorphous films with high porosity. Subsequently, for using particles smaller than 1 µm, the use of a solvent carrier is necessary for injecting the powder into the plasma. This solvent is evaporated when reaching the core of the plasma, while the solid particles melt, accelerate and impact on a substrate to form a coating [54]. Lee et al. [55] used a colloidal spray deposition of Pd particles in the range of 100–300 nm (using water with dispersing agent Darvan C as solvent) with additional heating during spraying at 110 °C. Their efforts resulted in porous Yttria-stabilized Zirconia (YSZ)supported Pd films after subsequent sintering (at 1160 °C for 3 h) with a controllable homogeneous thickness of about 5–11 µm and pores in the range of 3–7 µm. T. Boeltken et al. [56] tried to adapt a similar approach while using a solvent of higher viscosity with atmospheric plasma spraying (APS); 250–550 nm size particles were thus mixed in diethylene glycol monobutyl ether stabilized (DGME) with 10 wt.% ethyl cellulose while the suspension was heated at 65 °C for 24 h. Films of about 10 µm were formed on alumina and porous stainless steel with YSZ coatings used as diffusion barrier layer (produced also by APS). Both cases gave rise to reasonable hydrogen selectivity, revealing promising results. However, it does not yet meet the industrial application demands, due to challenges with the stability of the suspensions. Moreover, no information was given on the microstructure of the fabricated palladium membranes while using liquid suspension plasma spraying.

The selectivity of hydrogen and permeation of hydrogen through palladium membranes, alloyed or not, is a function of the microstructure and its changes due to prolonged heating during operation. Hence, this work focuses on forming a Pd coating with a submicronic microstructure with a large amount of LAGBs but suppressed SAGBs, using suspension plasma spraying (SPS) with nano-scale powder suspended in ethanol as well as using vacuum plasma spraying (VPS) using microscale powder. Both processes were developed to fabricate dense Pd membrane on porous supports. The main target was to see if these methods can offer the optimal grain size (100–500 nm) with LAGB for a stable membrane. In following work, this microstructure will be evaluated for hydrogen flux as well as selectivity at the working temperatures and under hydrogen atmosphere.

## 2. Materials and Methods

### 2.1. Membrane Supports

The metallic substrate used in this study was made of Crofer 22APU supplied by Thyssen VDM, Germany. The composition was 23% Cr, 0.45% Mn, 0.006% Ti, 0.1% La, <0.05% Al, <0.05% Si, 76.29% iron. The porous metallic substrate (with dimensions of 20.6 mm × 7.6 mm × 1 mm) was welded by laser to a dense frame of Crofer 22APU (48 mm × 35 mm × 1 mm). A layer of yttrium stabilized zirconia (YSZ) was coated on the porous metal with dip-coating technique. Detailed recipe for metal substrate preparation, coating of diffusion barrier layer is described in a previous study [11]. The porosity of porous Crofer 22APU was 32%, estimated with SEM images. Regarding the selection of the materials, Crofer 22APU is a ferritic steel that represents a thermal expansion coefficient of 12 × 10^−6^ K^−1^ at 20 °C to 11.6 × 10^−6^ K^−1^ at 800 °C. It is well compatible with other components, e.g., 8YSZ (10 × 10^−6^ K^−1^), and Pd (11.8 × 10^−6^ K^−1^) [56]. In order to minimize the effect of substrate quality on the coating of Pd, the same batch of sinter metal substrate was used. Moreover, around 30 samples were prepared and coated with 8YSZ (the same batch of coating suspension). Afterwards, the samples’ surfaces were observed and they were sorted based on defining class A (defect size < 1 µm), B, and C. In this study only, class A substrates were used for Pd coating.

### 2.2. Suspension Plasma Spraying

During this work, two different thermal plasma processes were examined for their suitability towards attaining the optimal microstructure of palladium coatings; either SPS using nanosized feedstock Pd powder injected as a suspension or VPS using microsized powder fed in as dry powder. Thermal plasma used in the current work was produced through a direct current (DC) arc giving a plasma jet exhibiting temperatures over 8000–14,000 K [57,58] and particle velocities in the range between 100 and 500 m.s^−1^ [59,60]. The temperature and momentum transfer from plasma to particle led to melting of the powder particles and their acceleration. These in-flight particle properties were controlled, along with the substrate temperature, to have the high bond strengths, low porosity and desired microstructure of the Pd coating in this work. Typically, the feedstock powders in thermal plasma are 10–100 µm [51,61]. In order to allow the use of nanoscale particles, particles were suspended and coatings were produced using SPS. Details can be found in References [62,63,64,65,66]. Nanosized metallic particles are pyrophoric and cannot be processed in plasma spraying as dry powder and therefore the use of a liquid makes this possible [56,61,62,67].

**Figure 1:**
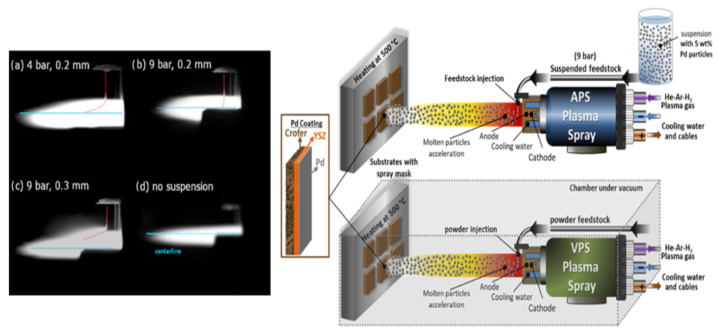
Left-hand side: CCD camera recordings of the injected water suspensions into the plasma flame (**a**) 4 bar, nozzle diameter of 0.2 mm, (**b**) 9 bar, nozzle diameter of 0.2 mm, (**c**) 9 bar, nozzle diameter of 0.3 mm, (**d**) plasma with no suspension as a reference. Right-hand side: schematics of the process for two techniques of APS and VPS in this study.

Figure 1 illustrates a schematic of the path the liquid suspension took in the SPS coating. For comparison, the VPS chamber is illustrated, showing how the process took place under vacuum, and using pure microstructure powder instead of liquid suspension. The gas flows were adjusted to increase the velocity and the temperature of the plasma accordingly, while reaching a comparable enthalpy of 9.48 KJ for the SPS and 11 KJ for the VPS.

For the SPS, experiments on the suspension supply and injection into the plasma jet were initially conducted while using pure solvent. It is necessary to adjust the injection parameters to ensure that the momentum of the particles is equal to the momentum of the plasma jet. In this way, the suspension bounces back from the plasma surface or crosses the plasma completely, and instead reaches the jet core [68]. Figure 1a–d summarizes the influence of the injection pressure used in the argon pressured tank (4 vs. 9 bar) for feeding the suspension to the jet and of the injection nozzle aperture used (0.2 mm vs. 0.3 mm). The trajectory of the suspension was indicated with a red line in all cases and compared to the entry point of the centerline. A 9-bar argon stream with nozzle size of 0.2 mm was found to be the most suitable injection condition.

The suspension/powder was then injected into the plasma (of a Sulzer-Metco TriplexPro 200 torch at a constant pressure of 9 bar. All plasma parameters, such as DC current, argon, helium and hydrogen flow were adjusted in order to optimize the treatment of the palladium particles to achieve a dense thin coating [69]. The Crofer 22 APU substrates coated with YSZ were clamped on the chamber holder and heated up to 500 °C during the entire process. The subsequent heating of the substrates permits better melting of the low concentration particles and therefore a denser microstructure at the final coating.

### 2.3. Palladium Powders

**Figure 2:**
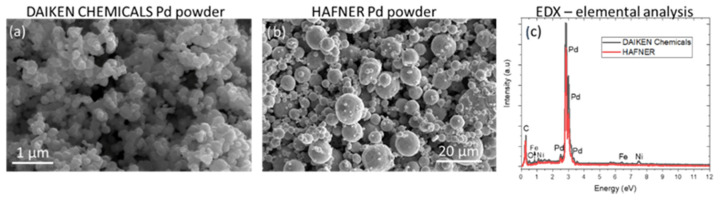
SEM images of the powders using 20 kV, 1.6 nA and ETD detector showing the average particle (**a**) Daiken chemicals Pd powder, (**b**) Hafner, and (**c**) EDX analysis of the powders showing their purity.

During this work, two different powders were used. A nanopowder from Daiken Chemicals for the suspensions prepared for the SPS coating and a micro-scale powder from Hafner for the VPS coating. The purification and particle size of the commercial palladium powders used were investigated and SEM images are shown in Figure 2a,b. An average particle size of 150 nm was obtained for the Daiken Chemicals with somewhat elongated areas and agglomerations. The particle size was required to be bigger in the Hafner powder since it was not used in a suspension and an average size of 8 µm was obtained with a variation of grains from 1–16 µm. ImageJ software was used to determine the particles size so that, for each powder, around 500 particles through different SEM images were analyzed. The corresponding elemental analysis by EDX for each powder is given in Figure 2c. Iron and nickel were shown as small impurities (less than 2 wt.%) in the case of the Daiken Chemicals powder. Both materials had a highly dense microstructure with few pores between the grains.

The viscosity of the suspension was adjusted to reduce the sedimentation of the particles, but at the same time avoid blocking of the nozzle when injecting. *TEGOMER DA 850* from *EVONIK* was chosen as an additive in pure ethanol. The viscosity of the final suspension (with 10 wt.% *Tegomer DA850*) was measured using RheoSense µVISC to be equal to ≈1.8 mPa.s at RT. The pH of the solution was measured to be 6.5 and its density 1.05 g.cm^−3^.

**Figure 3:**
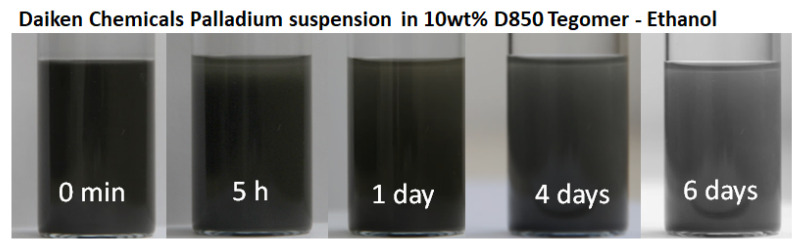
Photos taken of the suspension consisting of 5 wt.% of Daiken Chemicals palladium powder in 10 wt.% Tegomer in ethanol solution for different relaxing times.

The stability of the prepared suspension should meet the quality for SPS coating. Since the SPS may take only a few minutes, it was found to be satisfactory if the suspension stayed stable for couple of hours. To do so, photos were taken over a period of a week. The suspension was placed in an ultrasonic bath for 30 min before starting the process to resemble all the conditions of the SPS process. As shown in Figure 3, the suspension stayed significantly stable for a few hours allowing it to be used for SPS. First signs of segmentation appeared after 5 h; however, significant segmentation took place only after a few days. The remaining suspension after the SPS process could be recovered for further usage, if it was needed.

### 2.4. Coatings Characterization

Samples were investigated for screening the thickness and density of the coating with cross sectional imaging by a scanning electron microscopy-focus ion beam (SEM-FIB) dual beam microscope (Thermo Fisher SCIOS) with a gallium liquid metal field ionization source. The average thickness of Pd coatings was obtained by measuring the cross sections of the samples with SEM (Thermo Fisher SCIOS) analysis in both secondary electron (ETD) and backscattered (BSE) mode. A comparison of the coating stability before and after permeability tests was carried out. All electron microscopy images shown here were taken at 30 kV and 1.6 nA. Additional EDX analysis was carried out using EDAX TEAM analysis system from AMETEK material analysis division.

With the aim of investigating the crystallinity of the samples, a Siemens D5000 diffractometer was used, equipped with a Cobalt (Co) source: 6.9257 keV, λ = 1.7902 Å. A locked θ-2θ Bragg–Brentano geometry was used.

A Philipps CM200-FEG transmission electron microscope (TEM) was used at 200 kV for the bright and dark images, higher magnification images and selective area diffraction patterns (SADP). For TEM imaging, all samples were prepared by FIB lift outs. The preparation is explained in detail in Hadjixenophontos et al. [70]. For more detailed analysis of the grain orientation, boundaries and size EBSD from EDAX was used. The area of interest was cleaned with the ion beam used at 5 kV and 48 pA for 5 min in order to obtain a clear signal on the EBSD detector. The program OIM was used for the analysis of the data obtained using the TEAM software.

In order to study the grain changes and hydrogen influence on the morphology of the palladium coating, samples were exposed to pure hydrogen (10 bar) at 300 °C for a few days. For this, a cylindrical stainless-steel reactor from *BERGHOF* was used with a heating surrounding. The samples were later prepared by FIB lift-outs for TEM investigations.

## 3. Results and Discussion

**Figure 4:**
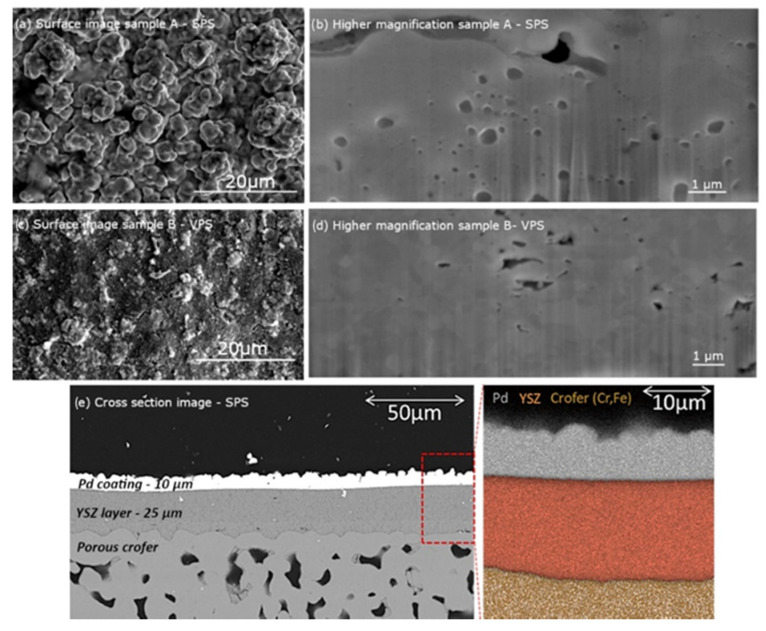
SEM micrographs of the samples using 30 kV, 1.6 nA. Secondary electron image of the surface showing the roughness of the layers for samples of SPS (**a**), and VPS (**c**). Cross-section view of the layers of SPS (**b**), and VPS (**d**). Backscattered detector cross section image ((**e**) left) and EDX elemental mapping analysis ((**e**) right) for SPS sample.

Since the aim of this study was to form a palladium coating with grain size in the range of 100–500 nm and with higher density of LAGBs for better hydrogen flux, the first investigations were focused on the crystallinity and morphology of the coatings. After plasma spraying, the samples were first imaged with SEM for surface and cross-sectional characterization. Figure 4a,b,e shows sample A prepared using the Daiken Chemical Pd powder suspension in SPS, and confirms the density of the sample and ≈10 µm thickness of the coating. The 8YSZ layer is also shown and its thickness was measured to be ≈25 µm. The porosity of the Crofer 22 APU substrate is visible while the EDX elemental mapping analysis in Figure 4e shows the different layers and their clear interfaces. Some differences are to be seen in the VPS coating where the pores appear to be fewer and a denser structure is visible in Figure 4c,d. The layer thickness for this sample was also estimated ≈10 µm. What is important to note is that the pores in both cases were of a diameter less than 1 µm and were isolated, not connected to each other.

Additionally, the coating’s surface was measured using a TURBO WAVE V7.8 profilometer by *HOMMELWERKE* at room temperature. An average roughness Ra = 5.27 µm and Ra = 1.39 µm for the SPS and the VPS samples respectively was obtained. Moreover, considering the substrates from the same batch, the porous Crofer and coated 8YSZ surface roughness were estimated 1.72 µm and <1 µm, respectively. The surface roughness of the Crofer surface might affect the quality of the 8YSZ, therefore, a two-step dip coating was used to produce a quite even and smooth surface for Pd coating. On the other hand, too smooth a surface might lead to poor adhesion between the Pd layer and 8YSZ.

**Figure 5:**
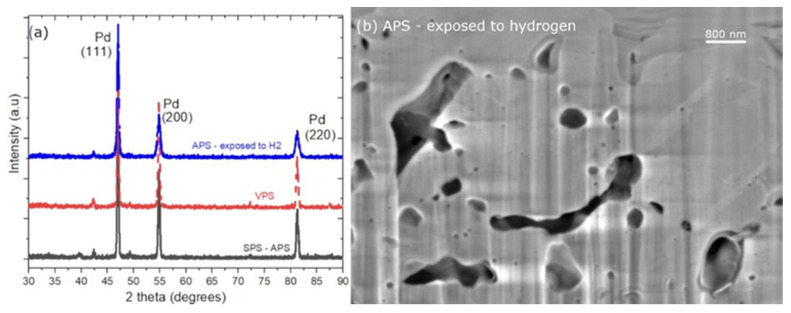
XRD patterns of the coated samples using a Co-source (**a**); samples of SPS-APS (black straight line), VPS (red dotted line), and APS exposed to H_2_ at 300 °C, 20 bars, for 75 h (blue dotted line). (**b**) SEM image of the APS sample showing remaining pores after treatment with H_2_ at 300 °C, 20 bars, for 75 h.

Additional characterization was carried out on both fabricated methods to examine the crystallinity of the palladium coating. The XRD spectra in Figure 5 indicated that the layers were fully crystalline and all palladium orientations were indicated with high intensities showing that there was no preferential orientation of the crystals. A small, inconsequential amount of impurities appeared at around 42°. Conclusively, suspension feedstock in SPS and powder feedstock in VPS are both capable of fabricating dense crystalline palladium membranes of a thickness of ≈10 µm.

As a comparison, the sample exposed to a 20-bar hydrogen atmosphere, at 300 °C for 75 h is shown in Figure 5. The XRD of the sample fitted to the previously as-prepared ones, with significantly broader peaks, showing signs of grain growth. The SEM micrograph in the image in Figure 5b is the same sample showing some pores still present in the sample after heat treatment. A more detailed analysis on the grains was carried out hereafter by TEM and EBSD.

**Figure 6:**
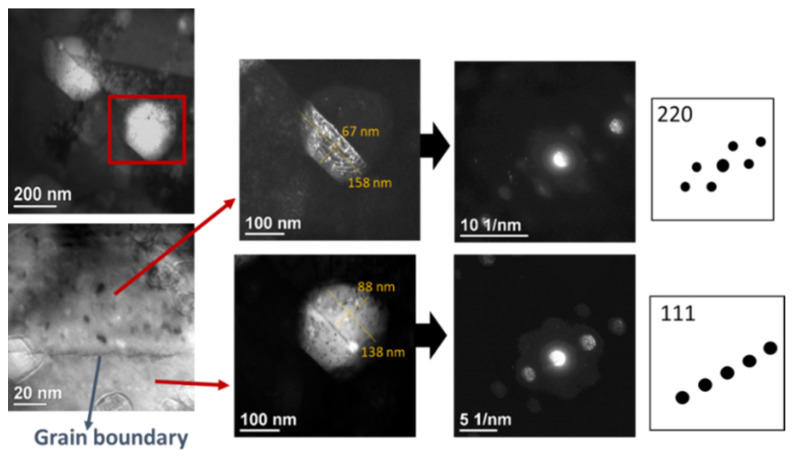
TEM analysis of two grains at two different orientations for APS sample without H_2_ treatment.

In order to investigate the grain boundaries of the palladium coatings before exposing the samples to hydrogen atmosphere, high resolution imaging of sample A—SPS was carried out, showing in this case, two grains combined to one particle. The grain boundary is indicated clearly at the line where the change in fast Fourier transformation (FFT) of both grains is obtained. In Figure 6, two grains are shown with their respective FFT showing that they have different orientations. This shows that careful analysis for the correct grain size is needed. For this, many dark field images were obtained, by tilting the beam to the different orientations, making sure every time that only one orientation was visible under the aperture. This same procedure was carried out for both samples.

**Figure 7:**
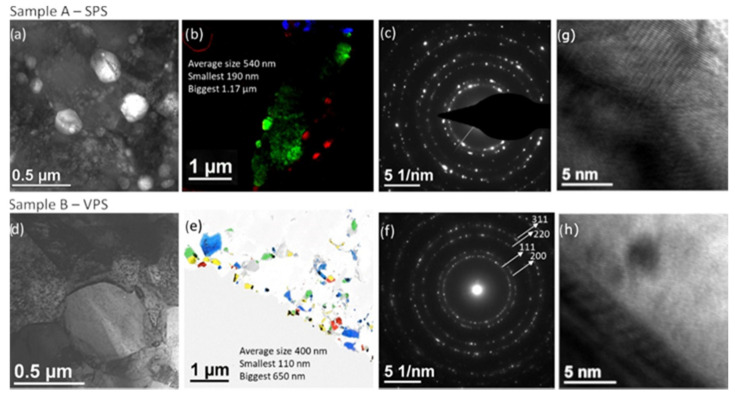
TEM bright field images of the samples (**a**,**d**), many dark field images of the coatings superposed with different colors showing the different orientations and the grain size (**b**,**e**), SADP of each sample showing in both cases the pure palladium (**c**,**f**), and HRTEM (**g**,**h**).

Figure 7 shows the bright field images of both samples before exposing them to hydrogen. Figure 7b,e for sample A—SPS and B—VPS, respectively, shows the dark field images representing the grains of different orientations. Each color represents a different orientation (different dark field image), showing in this way that there is a random orientation of the nanograins in both cases. In the case of the SPS sample, an average grain size of 540 nm was obtained with a wide range of grain size between 1.2 µm and 190 nm. While the VPS samples showed a slightly smaller grain size with an average of 400 nm (with grains between 650 nm and 110 nm). The SADP of both samples indicated the purity and crystallinity of the coatings indexed to pure palladium. Moreover, both samples showed the pure crystalline palladium in the HRTEM in Figure 7g,h.

**Figure 8:**
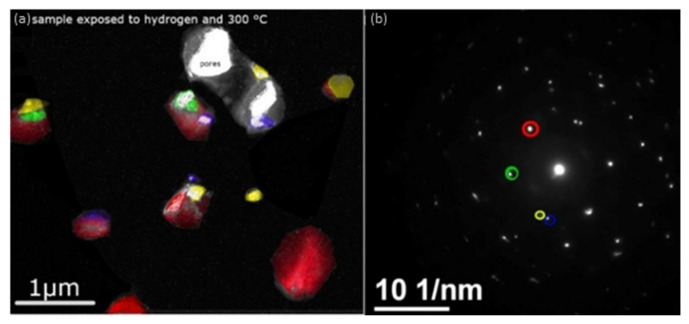
Superposition of dark field images of the reflection in circles for each color (**a**), Diffraction pattern with each color (shown in (**a**)) indicating the different grain orientation (**b**).

Special care at the crucial temperature of 300 °C in hydrogen atmosphere needs to be given for palladium coatings intended for hydrogen production and purification [71]. Since the α-to-β phase transition may occur at this temperature, both samples were exposed to 300 °C to see how the microstructure was affected. Figure 8 shows dark field images of the sample at different orientations. Figure 8b specifies at which reflection the grains belong to each indicating color. The red colored grains belong to the same reflection and appear to have significantly greater size. No significant change in the grain size is shown in this case (average grain size 610 nm), however, a delamination from the substrate occurred. As discussed earlier, the surface roughness of the 8YSZ layer is of great importance. The delamination after exposure to H_2_ could happen if the adhesion between the Pd layer and 8YSZ is poor. A crystal lattice expansion might happen, which, in turn, would result in a dilation causing compressive strain in the longitudinal direction [72,73]. Increasing the roughness of the 8YSZ surface might enhance the adhesion so that the Pd layer is not delaminated. This effect will be studied and addressed separately in the future.

It is worth mentioning that the pores were still visible in the sample with a diameter less than 1 µm while they were isolated without interconnection.

**Figure 9:**
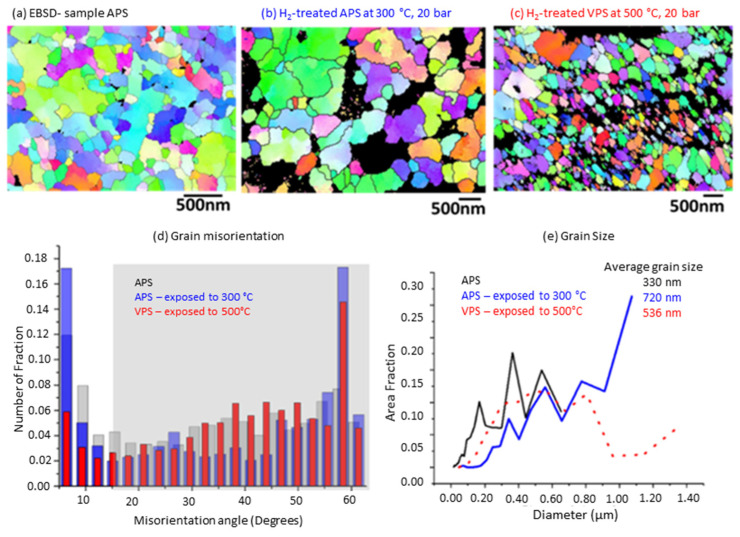
EBSD mapping of the samples: (**a**) as prepared after SPS-APS, (**b**) the SPS-APS sample heated at 300 °C, 20 bar of hydrogen for 72 h, (**c**) VPS samples exposed at 500 °C, 1 bar of hydrogen atmosphere, (**d**) misorientation angle between the grains, (**e**) the grain size distribution analysis from the respective EBSD map.

In order to see the misorientation of the grains in a larger area of the sample and to be able to characterize whether they had large or small angle grain boundaries, EBSD analysis was used. Figure 9 presents the EBSD mapping analysis for each sample, along with the grain misorientation and grain size. In the maps, each color shows a different orientation and, therefore, all maps show that there is no preferred orientation in the grains of the samples. The areas indicated in black are areas that due to roughness could not be measured by the detector. After increasing the temperature to 300 °C, the sample increased in grain size from an average of 330 nm to 720 nm. The orientation of the grains stayed, however, on the favorable side of the large-angle grain boundaries for hydrogen diffusion. In blue, in the graph in Figure 9d, one can see that all samples had a misorientation preferentially over the range of 15–60°. This indicates that the samples with SPS and VPS were mainly composed of LAGBs which were well maintained after heat treatment and exposure to hydrogen. This should assist in enhancing hydrogen diffusion through the coating.

## 4. Conclusions

Two manufacturing techniques including SPS and VPS were used for the production of a Pd coating in an attempt to obtain Pd membranes with grain size 0.1–1 µm, promoting LAGB while suppressing SAGB, which would allow the high selectivity and permeability of hydrogen. The SPS technique required a suspension consisting of Pd particles of 150 nm (nanosize) while a dry Pd powder with a particle size of 1–16 µm (microsize) was used for VPS. The substrates of Crofer 22 APU sinter metals (Ra = 1.72 µm, thickness 1 mm), double-coated with 8YSZ (Ra < 1 µm, thickness ≈ 25 µm) were used for the Pd coating. The implemented techniques of Pd coating led to an average grain size ranging around 500 and 600, probably allowing a good selectivity-permeation of hydrogen. Considering the phase transition of Pd, the samples were treated at 300 °C with a pure hydrogen stream and pressure of 20 bar for 72 h. This caused a change in the average grain size from 100–500 nm to 536 and 720 nm for VPS and SPS, respectively. Such a rather low thickness of Pd in comparison with commercial Pd foils might enhance the performance of a membrane reactor which was the ultimate aim of this study.

## Data Availability

Not applicable.
